# Prognostic Significance of Elevated UCHL1, SNRNP200, and PAK4 Expression in High-Grade Clear Cell Renal Cell Carcinoma: Insights from LC-MS/MS Analysis and Immunohistochemical Validation

**DOI:** 10.3390/cancers16162844

**Published:** 2024-08-14

**Authors:** Michał Kasperczak, Gabriel Bromiński, Iga Kołodziejczak-Guglas, Andrzej Antczak, Maciej Wiznerowicz

**Affiliations:** 1 Department of Urology, Poznań University of Medical Sciences, 61-701 Poznań, Poland; 2 International Institute for Molecular Oncology, 60-203 Poznań, Poland; 3 University Hospital of Lord’s Transfiguration, 61-848 Poznań, Poland

**Keywords:** clear cell, renal, carcinoma, gene expression, spectrometry, immunohistochemistry

## Abstract

**Simple Summary:**

This study explored the protein expression levels of UCHL1, SNRNP200, and PAK4 in clear cell renal cell carcinoma (CCRCC) using advanced proteomic techniques. We found that UCHL1 and SNRNP200 are significantly upregulated, and PAK4 is downregulated in high-grade CCRCC, and all are involved in critical cellular pathways linked to cancer progression and poor clinical outcomes. Specifically, UCHL1 is implicated in the Akt signaling pathway, SNRNP200 plays a role in RNA splicing and cell cycle regulation, and PAK4 is associated with cell proliferation and invasion. The enhanced expression of UCHL1 and SNRNP200, and decreased expression of PAK4 correlate with shorter progression-free survival in CCRCC patients, suggesting their potential as prognostic markers and targets for therapeutic intervention. This research contributes to the understanding of CCRCC’s molecular dynamics and opens new avenues for targeted treatments.

**Abstract:**

Recent advancements in proteomics have enhanced our understanding of clear cell renal cell carcinoma (CCRCC). Utilizing a combination of liquid chromatography-tandem mass spectrometry (LC-MS/MS) followed by immunohistochemical validation, we investigated the expression levels of UCHL1, PAK4, and SNRNP200 in high-grade CCRCC samples. Our analysis also integrated Reactome pathway enrichment to elucidate the roles of these proteins in cancer-related pathways. Our results revealed significant upregulation of UCHL1 and SNRNP200 and downregulation of PAK4 in high-grade CCRCC tissues compared to non-cancerous tissues. UCHL1, a member of the ubiquitin carboxy-terminal hydrolase family, showed variable expression across different tissues and was notably involved in the Akt signaling pathway, which plays a critical role in cellular survival in various cancers. SNRNP200, a key component of the RNA splicing machinery, was found to be essential for proper cell cycle progression and possibly linked to autosomal dominant retinitis pigmentosa. PAK4’s role was noted as critical in RCC cell proliferation and invasion and its expression correlated significantly with poor progression-free survival in CCRCC. Additionally, the expression patterns of these proteins suggested potential as prognostic markers for aggressive disease phenotypes. This study confirms the upregulation of UCHL1, SNRNP200, and PAK4 as significant factors in the progression of high-grade CCRCC, linking their enhanced expression to poor clinical outcomes. These findings propose these proteins as potential prognostic markers and therapeutic targets in CCRCC, offering novel insights into the molecular landscape of this malignancy and highlighting the importance of targeted therapeutic interventions.

## 1. Introduction

Clear cell renal cell carcinoma (CCRCC) accounts for approximately 3% of all cancer cases and is most frequently diagnosed in Western countries [1,2,3]. In the industrialized world, CCRCC is often diagnosed incidentally during imaging tests such as computed tomography scans, ultrasounds, or magnetic resonance imaging [4]. The classic symptoms—hematuria, flank pain, and palpable masses—are observed in only 10% of patients. CCRCCs are tumors of renal stem cell origin that typically arise in the tubular epithelium and proximal nephron. They account for 75% of RCC cases and are more likely to develop hematogenous metastases to the liver, lungs, and bones [4]. Genetic factors play a significant role, with up to 45% of CCRCC cases involving somatic mutations or deletions in the VHL gene.

Additionally, about 5% of CCRCC cases are due to hereditary VHL mutations linked to Von Hippel–Lindau disease and individuals with tuberous sclerosis gene mutations are more likely to develop bilateral RCC before age 46 [5]. Modifiable risk factors for RCC include food and alcohol consumption, obesity, poorly controlled hypertension, smoking, and occupational exposures [6]. Enhanced imaging protocols are essential to diagnose RCC at an earlier stage, alongside prevention programs that aim to improve survival rates and reduce disparities [7].

Patients with large primary tumors or those presenting with metastatic disease have observed the highest cancer-specific mortality rates [8]. However, the effectiveness of treatments for advanced or metastatic CCRCC remains limited, prompting extensive molecular research to develop targeted therapies [9]. Understanding the molecular complexities in advanced/metastatic CCRCC lays the groundwork for developing targeted therapies and refining treatment strategies. Recently, significant efforts were directed toward the proteomic profiling of CCRCC to identify protein biomarkers [10,11]. These studies typically involve comparing protein profiles using The Cancer Genome Atlas (TCGA) data or contrasting tumor tissue with normal tissue [12]. Modern proteomic techniques, such as tandem mass spectrometry, and innovative proteogenomic approaches that combine reference and sample-specific genomes are enhancing our understanding of cancer at the molecular level [13,14,15].

Essential proteins such as Ubiquitin C-terminal hydrolase 1 (UCHL1), which recycles ubiquitin from degraded proteins and facilitates protein degradation, are highly expressed in the nervous system and various carcinomas [16,17]. The role of the spliceosome, particularly the U5 snRNP’s 200 kDa helicase encoded by the SNRNP200 gene, is also crucial, given its association with tumor aggressiveness in prostate cancer [18,19]. Furthermore, the role of p21-activated kinases (PAKs), serine/threonine protein kinases, and downstream effectors of the Rho GTPases underscores their significance in cell proliferation, angiogenesis, tumorigenesis, and metastasis [20,21].

Therefore, this study aimed to determine the expression levels of UCHL1, SNRNP200, and PAK4 proteins using immunohistochemistry (IHC) assay to elucidate the impact of molecular alterations driving phenotypic changes and to delineate the mechanisms of CCRCC pathobiology and progression for prospective exploration of personalized precision-based clinical care.

## 2. Materials and Methods

### 2.1. Patients and Cohorts

**Figure 1:**
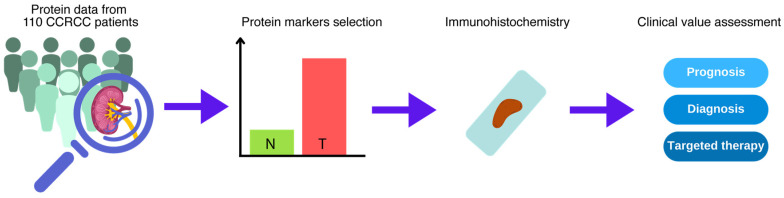
Study design: key steps of the study. Acquisition of protein data from the CPTAC discovery CCRCC cohort dataset and subsequent analysis of protein expression of tumor (T) and normal adjacent tissue (N) to select candidate protein markers. The selected protein markers were next subjected to immunohistochemical analysis. Assessment of the clinical value of the selected proteins for prognosis, diagnosis, and targeted therapy for CCRCC patients.

This study leverages a CCRCC discovery cohort provided by the Clinical Proteomic Tumor Analysis Consortium (CPTAC), consisting of 110 treatment-naive CCRCC cases alongside 84 paired-matched normal adjacent tissue (NAT) samples, as detailed in Clark et al. [15]. Utilizing publicly available LC-MS/MS protein expression data and a thorough literature review, we identified and selected critical protein markers for further investigation and immunohistochemical (IHC) analysis (Figure 1). The proteomic data, sourced from the publicly available CPTAC dataset, enabled an in-depth examination of protein markers contributing to CCRCC development and metastasis. Due to their biological significance, we selected proteins with increased expression (SNRNP200) and decreased expression (PAK4 and UCHL1) in tumors relative to normal adjacent tissues. The expression of the validated markers was then correlated with clinical data through IHC analysis, integrating proteomic data acquisition, marker validation, and clinical correlation into a methodological framework that enhances our understanding of CCRCC pathobiology and progression and informs potential clinical applications.

The validation cohort included 52 ccRCC samples from patients aged 31 to 84, with 23 participants also enrolled in the CPTAC discovery study. Comprehensive demographic and tumor characteristic data—age, gender, race, grade, and stage—were systematically documented and are summarized in Supplementary Table S1. Of these patients, 26 developed metastasis while another 26 did not during the five-year follow-up period. This investigation focused on adult CCRCC tumors confirmed histopathologically. Ethical approval was secured following CPTAC guidelines and the study excluded patients who had undergone systemic treatment or had additional cancers in the past 12 months.

### 2.2. Immunohistochemistry and Pathology Evaluation 

IHC analysis was conducted using the Dako Autostainer Link 48, following preprogrammed staining protocols and by employing the EnVision visualizing kit (Cat No. K800221-2, Dako, Agilent Technologies Inc., Carpinteria, CA, USA). Tissue samples were collected retrospectively, fixed in 10% neutral buffered formalin for 24 h, and then embedded in paraffin. Sections, 4 microns in thickness, were cut from formalin-fixed paraffin-embedded (FFPE) blocks and placed on positively charged slides. Antigen retrieval was performed using Dako Target Retrieval Solution, High pH (Cat No. S2367), in a PT Link Pre-Treatment Module (Dako, Agilent Technologies Inc.) at 97 °C for 20 min. Following antigen retrieval, the sections were incubated with primary antibodies for 30 min at room temperature. For the clinical validation cohort, three primary antibodies were utilized, as detailed in Table 1 The IHC-stained slides were manually evaluated by three independent pathologists using the H-score methodology to assess the results. The H-score is a semi-quantitative measure that combines both the intensity and the percentage of positive staining cells, resulting in a score ranging from 0 to 300. It is calculated as follows:

H-score = (% of cells stained at intensity 1 × 1) + (% of cells stained at intensity 2 × 2) + (% of cells stained at intensity 3 × 3)

In this formula:

Intensity 1 indicates weak staining.

Intensity 2 indicates moderate staining.

Intensity 3 indicates strong staining.

The percentages represent the proportion of cells exhibiting each staining intensity. By summing the products of the intensity scores and their respective percentages, the H-score provides a comprehensive assessment of the extent and intensity of IHC staining within a given specimen [22]. 

The cohort used to assess PAK4, SNRNP200, and UCHL1 expression via IHC analysis included 52 CCRCC formalin-fixed paraffin-embedded (FFPE) samples. IHC specimens were categorized into groups for downstream analyses based on H-score ranges. Patients with IHC scores above the calculated cutoff were placed in the High IHC score group, while those with scores below the cutoff were assigned to the Low IHC score group (Table 2). For each protein, the average of all H-scores from the 52 IHC slides was calculated to determine the cutoff value for categorizing patients into High IHC score and Low IHC score groups. Notably, one UCHL1 sample was excluded from this analysis.

### 2.3. Digital Image Acquisition and Storage

All IHC slides were digitally scanned with the automated ScanScope AT Turbo whole slide scanner (Aperio/Leica Microsystems, Vista, CA, USA) using 20× magnification. Digital images of the IHC slides were saved in .svs format and reviewed using ImageScope software (12.3.3 version, Aperio, Vista, CA, USA) to facilitate a high-quality examination by expert pathologists. The digital images were accessed through a password-protected Synology Rack Station server (RS18017xs+).

### 2.4. Statistical Analysis

Protein abundance differences between normal and tumor tissues in CCRCC were computed using a non-parametric Wilcoxon rank-sum test. For comparison of IHC scores obtained for the High IHC score group and the Low IHC score group of the validation cohort, the Wilcoxon rank-sum test was performed. Differences in protein abundance between groups were also assessed using the Mann–Whitney U test. Overall survival (OS) and progression-free survival (PFS) analyses for the validation cohort were performed by dividing the validation cohort into two groups, the High IHC score group and the Low IHC score group (separately for each protein target), which were then compared using the Kaplan–Meier method and log-rank (Mantel–Cox) test. Hazard ratios (HR) and their 95% confidence intervals (CI) were calculated to assess the impact on survival outcomes. All data were analyzed using GraphPad Prism 10 software. A *p*-value <0.05 was used to determine statistical significance in all analyses.

### 2.5. Reactome Pathway Enrichment Analysis

To gain deeper insights into the cellular processes and tumor mechanisms associated with PAK4, SNRNP200, and UCHL1, we conducted a Reactome pathway enrichment analysis. This analysis aimed to identify the most significant Reactome pathways enriched for each protein, thereby elucidating their functional implications in CCRCC.

Protein expression data for PAK4, SNRNP200, and UCHL1 were obtained from the publicly available CPTAC CCRCC dataset. The Reactome pathway enrichment analysis was performed using the Reactome database, which provides a comprehensive collection of biological pathways. The analysis was conducted with the Reactome Pathway Analysis tool, where the protein expression data were input to identify pathways significantly associated with PAK4, SNRNP200, and UCHL1. A statistical significance threshold of *p* < 0.05 was applied to determine the enriched pathways, ensuring that the identified pathways were highly likely to be biologically relevant.

## 3. Results

### 3.1. Protein Expression Values in CPTAC CCRCC

**Figure 2:**
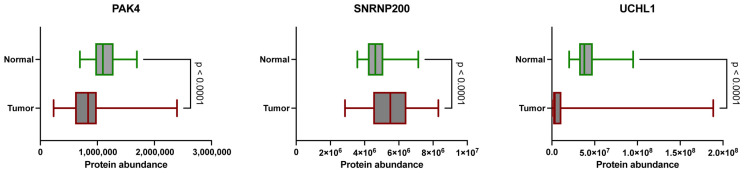
Protein abundance value distribution between normal adjacent tissue and tumor samples for PAK4, SNRNP200, and UCHL1 as measured by LC-MS/MS. Boxes outlined in green represent normal adjacent tissue samples and boxes outlined in red represent tumor samples (*p* < 0.0001).

Among the analyzed proteins, PAK4, SNRNP200, and UCHL1, SNRNP200 exhibited significantly increased abundance in CCRCC tissues compared to NATs (Figure 2). Conversely, PAK4 and UCHL1 showed elevated levels in NATs compared to tumor samples. These findings were statistically significant (*p* < 0.0001), as determined by the Mann–Whitney U test.

### 3.2. Immunohistochemical Analysis of UCHL1, SNRNP200, and PAK4 Reveals Protein Candidates for Further Correlation with Patients’ Clinical Outcomes

**Figure 3:**
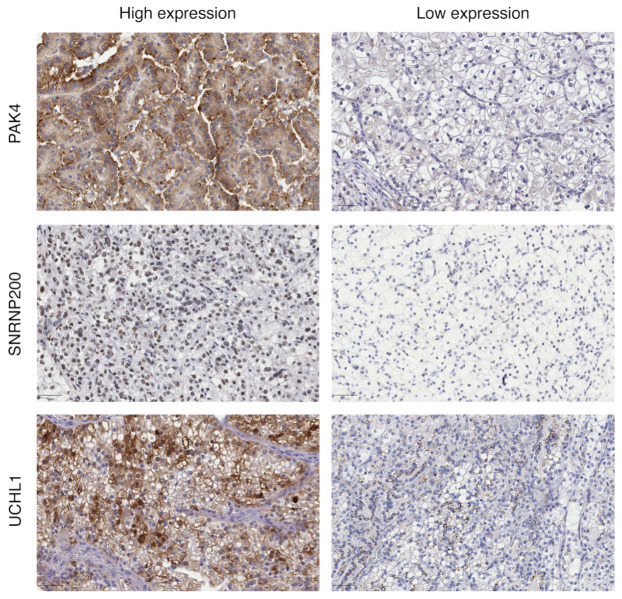
Immunohistochemical analysis showing immunostaining patterns in representative tumor sections for PAK4, SNRNP200, and UCHL1 high and low expression in the CCRCC validation cohort. Brown color indicates positive immunoreaction and blue indicates negative immunoreaction.

The immunohistochemical analysis revealed distinct expression patterns for PAK4, SNRNP200, and UCHL1 in CCRCC cells (Figure 3). PAK4 was expressed in both the cytoplasm and the nucleus of CCRCC cells. SNRNP200, on the other hand, was predominantly expressed in the nucleus of CCRCC cells. UCHL1 was found to be expressed in multiple cellular compartments, including the cytoplasm, membrane, and nucleus of CCRCC cells. After completing the IHC staining, the resulting scores for each protein were analyzed through a pathology evaluation as outlined in the Section 2. Subsequently, the IHC scores were correlated with survival data of CCRCC patients, aiming to elucidate the potential prognostic significance of PAK4, SNRNP200, and UCHL1 expression levels in the context of patient outcomes, especially metastasis.

### 3.3. PAK4, SNRNP200, and UCHL1 Protein Expression as Measured by IHC 

**Figure 4:**
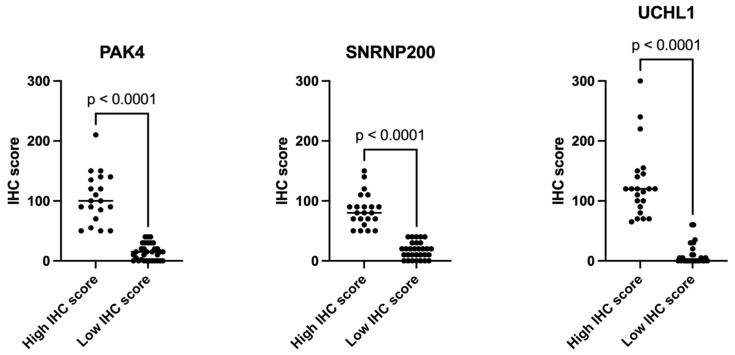
Distribution of IHC scores obtained for PAK4, SNRNP200, and UCHL1. The scatter plots demonstrate the variation in scores between “high score” and “low score” groups across the different proteins. All plots show statistically significant differences (*p* < 0.0001), indicating pronounced disparities in IHC scores between the two groups. Notably, for UCHL1, the high score group exhibits a wider range of scores, indicating varying expression levels among individuals, unlike PAK4 and SNRNP200, where the high score groups are more tightly clustered without significant outliers.

Following the IHC analysis, we analyzed the scores for PAK4, SNRNP200, and UCHL1. As illustrated in Figure 4, distinct distribution patterns were observed in the CCRCC validation cohort. For all proteins, the pronounced disparities between the two groups are statistically significant, indicating a pronounced dichotomy in their expression levels. However, while PAK4 and SNRNP200 had no significant outliers, highlighting the uniformity of expression across each subgroup, in the High IHC score group of the UCHL1 protein, notable outliers exist, indicating variability in its expression among certain patients. 

### 3.4. PAK4, SNRNP200, and UCHL1 Protein Expression Correlated with Patients’ Clinical Outcomes

**Figure 5:**
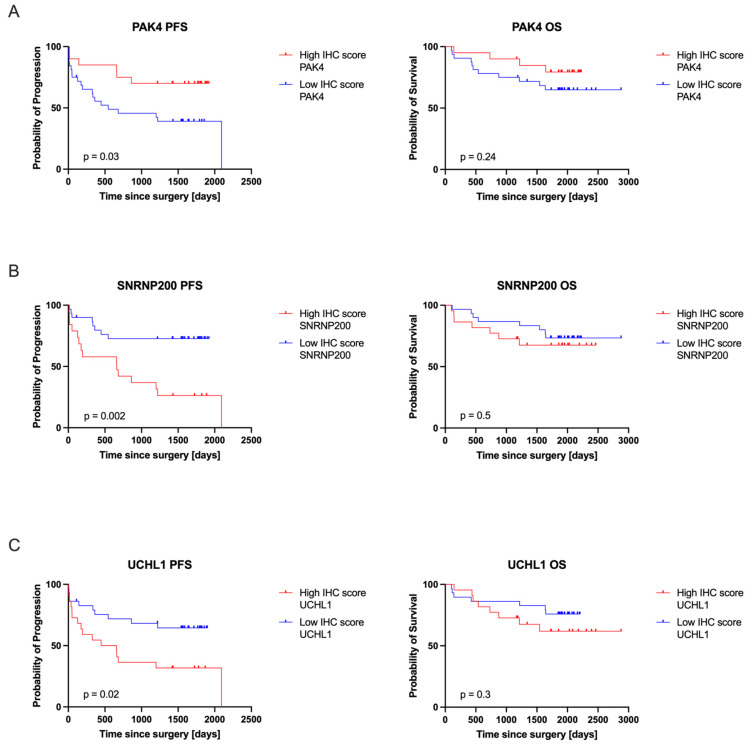
IHC scores for PAK4 (**A**), SNRNP200 (**B**), and UCHL1 (**C**) were analyzed to determine their correlation with patients’ clinical outcomes, specifically PFS and OS. Patient outcomes were compared by categorizing them according to the highest and lowest IHC scores, providing a clear comparison between these two groups. Both OS and PFS were meticulously tracked over a five-year follow-up period.

The relationship between the expression levels of target proteins, as determined by IHC, and their clinical relevance was evaluated using survival analyses. This assessment involved comparing patient outcomes based on the highest and lowest IHC scores, as shown in Figure 5. Both OS and PFS were monitored over a five-year follow-up period. Moreover, the impact of protein expression measured by IHC on patients’ PFS and OS in the validation cohort is presented in Table 3. Our findings indicate that higher expression levels of SNRNP200 and UCHL1 are associated with poorer PFS (*p* = 0.002 and *p* = 0.02, respectively) and increased risk of progression (HR = 3.35 and 2.39, respectively), while lower expression of PAK4 correlates with poorer PFS (*p* = 0.03) and increased risk of disease progression (HR = 2.57). It should be noted that in our study, an HR > 1.0 for PAK4 reflects an unfavorable role, where lower expression levels correlate with a higher risk of disease progression. The expression levels of PAK4, SNRNP200, and UCHL1 did not significantly affect OS (*p* > 0.05). Furthermore, PAK4 shows an HR of 1.96, SNRNP200 an HR of 1.41, and UCHL1 an HR of 1.69, all indicating potential increases in mortality risk, though none are statistically definitive due to CIs that span or approach 1. This suggests that while these proteins may influence survival, their effects are not conclusively significant.

### 3.5. Identification of the Top Significantly Enriched Reactome Pathways Associated with PAK4, SNRNP200, and UCHL1

**Figure 6:**
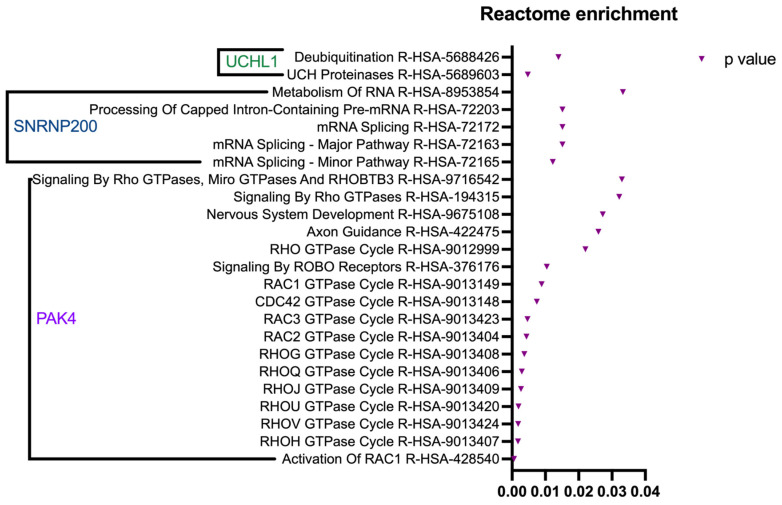
Significant Reactome Enrichment Pathways for PAK4, SNRNP200, and UCHL1. Visual representation of statistically significant (*p*-value < 0.05) enriched terms associated with each protein.

To gain deeper insights into the cellular processes and tumor mechanisms associated with PAK4, SNRNP200, and UCHL1, we conducted a detailed analysis of the most significant Reactome enrichment pathways, as illustrated in Figure 6. The Reactome enrichment analysis was specifically designed to reveal the functional implications associated with each of the three proteins. The analysis identified statistically significant enriched terms for each protein, using a significance threshold of *p* < 0.05, thereby underscoring the distinct biological pathways influenced by each protein. 

The Reactome enrichment analysis for UCHL1 identified two significantly enriched pathways, “Deubiquitination” and “UCH Proteinases”, indicating UCHL1’s critical roles in protein ubiquitination processes. For SNRNP200, the analysis highlighted its significant involvement in mRNA splicing, as evidenced by the enriched pathways associated with this fundamental cellular mechanism. For PAK4, the Reactome enrichment analysis demonstrated significant enrichment in pathways associated with GTPase activity, specifically highlighting RHO GTPase and RAC GTPase pathways. Additionally, the analysis revealed significant involvement of PAK4 in pathways related to the nervous system.

## 4. Discussion

Our investigation of CCRCC patients employed LC-MS/MS analysis from a publicly available CPTAC dataset, encompassing a cohort of 110 tumor samples. This analysis revealed a noteworthy upregulation in the expression levels of SNRNP200 and downregulation of UCHL1 and PAK4 proteins in the CPTAC tumor samples compared to normal samples. We further validated these findings using immunohistochemistry (IHC) in an independent cohort of CCRCC patients’ FFPE samples, including high-grade CCRCC samples. Interestingly, in high-grade CCRCC, the protein expression levels of UCHL1 and SNRNP200 were found to be elevated, while PAK4 expression was decreased. Importantly, we identified a significant correlation between the particular expression profiles of these proteins and shortened PFS in patients with high-grade CCRCC. This suggests that UCHL1, SNRNP200, and PAK4 could serve as valuable prognostic markers, offering new insights into the progression and prognosis of this aggressive cancer subtype. In the context of the existing literature, the observed upregulation of UCHL1 and SNRNP200 and the downregulation of PAK4 provide a novel dimension to the molecular landscape of high-grade CCRCC, offering insights that complement and extend the current understanding of this aggressive cancer subtype.

In our study, IHC analysis identified distinct expression patterns for PAK4, SNRNP200, and UCHL1 in CCRCC cells. UCHL1 was localized in the cytoplasm, membrane, and nucleus, highlighting its multifunctional roles in cellular regulation [17]. The results we obtained from the Reactome enrichment analysis for UCHL1 identified ‘Deubiquitination’ and ‘UCH Proteinases’ as significantly enriched pathways, illuminating the involvement in crucial ubiquitin-mediated processes and protein degradation pathways. These pathways are vital for maintaining cellular homeostasis and play significant roles in cancer development and progression. The link between UCHL1 and deubiquitination underscores its significance in cellular functioning but also positions it as a potential therapeutic target [23]. This finding calls for further research into the specific mechanisms through which UCHL1 influences tumorigenesis.

The IHC scores obtained for UCHL1 showed a bimodal distribution, with significant outliers in the High IHC score group, indicating variation in expression among some patients. This variability suggests heterogeneity within the CCRCC patient population, potentially reflecting distinct molecular subtypes or clinical phenotypes. UCHL1 is differently expressed across tissues and tumors. While it is among the most abundant proteins in the brain, it has been identified as either an oncogene or a tumor suppressor in different carcinomas, such as nasopharyngeal, gastric, colorectal, and ovarian [24,25,26]. In the kidney, UCHL1 is highly expressed in distal tubules and minimally in collecting ducts [27]. Interestingly, it was hypothesized that UCHL1 expression may be actively repressed during the initial stages of tumorigenesis, with its re-expression in later stages potentially serving as a reliable indicator of metastatic disease [28]. This observation aligns with recent research showing higher frequencies of UCHL1 methylation in primary CCRCC compared to normal kidney epithelium, suggesting a complex role of UCHL1 in the progression of renal cancer [29].

UCHL1 overexpression in CCRCC was associated with a more aggressive potential and metastatic cancer phenotype and, in some cases, a poor prognosis for the patient [29,30,31]. Our study confirmed these findings, as high expression of UCHL1 was associated with worse PFS. Seliger B et al. showed that the methylation of the UCHL1 DNA promoter leads to UCHL1 silencing in both ccRCC cell lines and primary lesions, mainly of clear cell subtype. This is corroborated by the observation that both the transcriptional and translational expression patterns exhibit a link with the methylation status of the CpG islands in the UCHL1 DNA promoter [30]. UCHL1 promotes cell survival and growth in B-cell malignancies, significantly influencing the Akt signaling pathway, crucial for cell proliferation and survival in cancers like lymphoma [32,33,34]. As a critical component of the proteasome targeting the ubiquitin-dependent protein degradation pathway, UCHL1 is involved in key cellular functions such as proliferation, cell cycling, apoptosis, and intracellular signaling [35], all of which are often disrupted in cancer [36]. Ummnanni R et al. described UCHL1 downregulation in prostate cancer and its tumor suppressor function in LNCaP prostate cancer [37]. Their analysis showed significantly lower levels of UCHL1 in cancerous prostate tissue compared to nearby non-cancerous tissue [37]. 

Current therapeutic approaches targeting UCHL1 in CCRCC include investigating its role in tumor growth and resistance to treatments like bevacizumab. UCHL1 mediates the deubiquitination of KDM4B, stabilizing KDM4B protein levels, which then promotes VEGFA expression and angiogenesis. UCHL1’s interaction with KDM4B is crucial for CCRCC malignancy and targeting UCHL1 has been shown to suppress tumor growth and enhance sensitivity to bevacizumab [38]. 

Small nuclear ribonucleoprotein U5 subunit 200 (U5 snRNP200) is a conserved and essential component of the RNA splicing machinery, primarily localized in the nucleus, as confirmed by our IHC results. However, although U5 snRNP200 functions in the nucleus, previous research indicates that, in its absence, the U5 snRNP complex can form in the cytoplasm, subsequently assembling into the larger U5 snRNP complex within the nucleus [39]. In terms of tissue-specific expression, SNRNP200 exhibits varied levels across different tissues; in the kidney, for example, expression is low in glomerular cells and medium in tubular cells [27]. In hepatocellular carcinoma (HCC), its expression is significantly elevated and positively correlates with tumor size, serum AFP levels, organ invasion, and poor survival outcomes [40,41]. Furthermore, SNRNP200 is implicated in the aggressiveness of prostate cancer tumors, highlighting its role in tumor pathology and suggesting its potential as a target for therapeutic intervention [19,42]. Mutations in SNRNP200 have been linked to conditions like autosomal dominant retinitis pigmentosa and single-cell RNA-seq studies in acute myeloid leukemia (AML) cells have demonstrated a strong association between cell surface expression of U5 snRNP200 and components of the antiviral innate response pathway [43,44].

The IHC scores for SNRNP200 are sharply divided between high and low, revealing a pronounced dichotomy in expression levels that could impact the clinical management of cancer. This distinction offers essential insights into patient stratification, prognosis, and therapeutic approaches. Furthermore, Reactome enrichment analysis has shown that SNRNP200 is notably enriched in pathways related to mRNA splicing, underscoring its crucial role in regulating splicing events that are vital for tumorigenesis. Dysregulation of mRNA splicing is recognized as a cancer hallmark, contributing substantially to tumor heterogeneity and progression by altering gene expression patterns [45]. SNRNP200 is a crucial part of the spliceosome and plays an important role in mRNA splicing in CCRCC [46]. Targeting splicing issues in this cancer could offer potential therapeutic benefits. The overexpression of SNRNP200 in prostate cancer has been associated with tumor aggressiveness [19]. This study is the first report demonstrating that, in humans, SNRNP200 is highly correlated with PFS in CCRCC. The identification of specific splicing isoforms associated with CCRCC may provide valuable biomarkers for disease diagnosis and prognosis.

Current therapeutic approaches for targeting SNRNP200 in CCRCC are still emerging but there is significant potential based on findings from studies in other cancers, particularly AML. In AML, targeting SNRNP200 with engineered antibodies has demonstrated potential by enhancing the immune-mediated clearance of cancer cells and avoiding off-target effects [44]. Combining SNRNP200-targeted therapies with existing treatments, such as immune checkpoint inhibitors, could improve outcomes by disrupting cancer cell processes and boosting immune responses. Further research is needed to fully explore its therapeutic potential in CCRCC.

In our study, PAK4 was expressed in both the cytoplasm and the nucleus of CCRCC cells, indicating its potential involvement in intracellular signaling pathways. PAKs, often overexpressed or amplified in various human cancers, play a critical role in cell transformation, making them promising targets for therapeutic intervention [47,48,49,50]. PAKs are involved in regulating various cellular functions such as cell proliferation, angiogenesis, tumorigenesis, and metastasis [51]. Specifically, in RCC, PAK1 has been shown to regulate cell proliferation, invasion, anchorage-independent growth, apoptosis, and resistance to 5-fluorouracil [52].

In terms of clinical implications, the elevated expression of PAK4 is associated with early recurrence and reduced survival rates in patients with nonmetastatic CCRCC, particularly those classified as low-risk following nephrectomy [53]. Interestingly, our IHC results showed that PAK4 expression was decreased in high-grade CCRCC, showing a strong association with metastasis appearance. Evidence suggests that PAKs play a crucial role in the biology of RCC, indicating an association between total PAK expression and aggressiveness and poor survival [54]. Our study assessed the impact of PAK4 expression in surgically treated RCC and found a significant association with PFS in CCRCC, although PAK4 was not identified as a top-ranked gene in this context by Linehan et al. [55]. This aligns with findings from Park et al., which indicated that the PAK4-Slug axis might exacerbate the prognosis of prostate cancer by promoting the epithelial–mesenchymal transition [56]. Additionally, Liu et al. reported that high PAK4 expression is linked to early recurrence and poor survival in patients with non-metastatic clear cell RCC post-surgery [53]. However, high PAK4 expression has been identified as a poor prognostic factor in two specific patient subgroups: those with early T stage (T1–2) disease and those with low Fuhrman grade (grade 1–2), underscoring the complex role of PAK4 in RCC and highlighting its potential as a target for personalized medicine approaches [53]. Although many studies show high levels of PAK4 in RCC, there are also reports of varying expression levels depending on the study design and patient groups. Different trends in studies highlight the complexity of cancer biology and the need for careful interpretation. We think this explains the differences and emphasizes the importance of considering context in biomarker studies.

Current therapeutic approaches for treating CCRCC are increasingly exploring the potential of targeting PAK4. Several small molecule inhibitors targeting PAK4, such as ATP-competitive inhibitors like PF-3758309 and LCH-7749944, have shown promising effects in different cancers in preclinical models by inhibiting PAK4 activity and subsequently reducing tumor growth and metastasis [57,58,59]. However, the non-specificity and off-target effects of these inhibitors have limited their clinical success. Recently, the development of proteolysis targeting chimeras (PROTACs) targeting PAK4 has emerged as a novel therapeutic strategy. These PROTACs selectively degrade PAK4, offering a more targeted approach that may overcome the limitations of classical inhibitors. Early studies suggest that PAK4-targeting PROTACs not only inhibit tumor growth but also enhance immune responses, potentially improving outcomes when combined with existing therapies like immune checkpoint inhibitors. While these findings are promising, further investigation is needed to determine the safety, efficacy, and clinical utility of PAK4-targeted therapies in ccRCC.

## 5. Conclusions

In conclusion, our comprehensive analysis utilizing LC-MS/MS and followed by immunohistochemistry has unveiled a significant elevation in UCHL1 and SNRNP200 and a decrease in PAK4 protein expression levels in high-grade clear CCRCC. The rigorous validation of these findings underscores the robustness of our results. Moreover, the observed correlation between increased expression of UCHL1 and SNRP200 and decreased expression of PAK4 and shorter PSF in high-grade CCRCC patients suggests prognostic relevance. This study not only expands our understanding of the molecular factors associated with CCRCC progression but also opens avenues for further research into targeted therapeutic interventions aimed at mitigating the adverse outcomes associated with UCHL1, SNRNP200, and PAK4 expression in this context.

## 6. Study Limitations

This study has several limitations that should be acknowledged. First, the number of samples used for immunohistochemical (IHC) validation was relatively small, consisting of 52 CCRCC cases with formalin-fixed paraffin-embedded (FFPE) blocks. The limited sample size may affect the generalizability of the findings and the robustness of statistical analyses. Future studies with larger cohorts are needed to validate these results and strengthen the conclusions drawn from this research.

Second, the IHC analysis was conducted using research antibodies rather than in vitro diagnostic (IVD) antibodies. The use of research antibodies was necessitated by the unavailability of suitable IVD antibodies for the targeted proteins. While research antibodies can provide valuable insights, their performance may vary and their specificity and sensitivity might not be as rigorously validated as IVD antibodies. This limitation should be considered when interpreting the results and the findings should be corroborated with additional studies using IVD antibodies when they become available.

## Figures and Tables

**Table 1:** Primary antibodies used for immunohistochemical analysis.

Antibody	Source	Identifier	Clone	Dilution
Rabbit polyclonal anti-UCHL1	Atlas Antibodies, Stockholm, Sweden	Cat# HPA005993, RRID:AB_1858560	Polyclonal	1:3000
Rabbit polyclonal anti-SNRNP200	Atlas Antibodies, Stockholm, Sweden	Cat# HPA029321, RRID:AB_10604096	Polyclonal	1:225
Mouse monoclonal anti-PAK4	ThermoFisher Scientific, Waltham, MA, USA	Cat# MA5-26859, RRID:AB_2723443	OTI1C7	1:150

**Table 2:** Classification of IHC specimens into groups for downstream analyses based on H-score ranges.

Protein	H-Score Range—High IHC Score Group	H-Score Range—Low IHC Score Group	Reaction
PAK4	50–210	0–40	Cytoplasmic, membranous
SNRNP200	50–150	0–40	Nuclear
UCHL1	65–300	0–60	Cytoplasmic, membranous, nuclear

**Table 3:** Impact of protein expression measured by IHC on patients’ survival in CCRCC validation cohort. HR and 95% CIs for PAK4, SNRNP200, and UCHL1, evaluating their impact on PFS and OS.

	Progression-Free Survival (PFS)		Overall Survival (OS)	
	Hazard Ratio (HR)	95% CI of HR	Hazard Ratio (HR)	95% CI of HR
PAK4	2.57	1.181–5.569	1.96	0.7027–5.476
SNRNP200	3.35	1.427–7.842	1.41	0.498–3.995
UCHL1	2.39	1.092–5.240	1.69	0.602–4.738

## Data Availability

All data on which the manuscript was based, if not already included in its text, are available from the corresponding author on a reasonable request.

## Supplementary Materials

The following supporting information can be downloaded at https://www.mdpi.com/article/10.3390/cancers16162844/s1, Table S1. Characteristics of patients included in the study.

## References

[1] Ferlay J., Colombet M., Soerjomataram I., Dyba T., Randi G., Bettio M., Gavin A., Visser O., Bray F. (2018). Cancer Incidence and Mortality Patterns in Europe: Estimates for 40 Countries and 25 Major Cancers in 2018. Eur. J. Cancer.

[2] Padala S.A., Barsouk A., Thandra K.C., Saginala K., Mohammed A., Vakiti A., Rawla P., Barsouk A. (2020). Epidemiology of Renal Cell Carcinoma. World J. Oncol..

[3] Bukavina L., Bensalah K., Bray F., Carlo M., Challacombe B., Karam J.A., Kassouf W., Mitchell T., Montironi R., O’Brien T. (2022). Epidemiology of Renal Cell Carcinoma: 2022 Update. Eur. Urol..

[4] DeCastro G.J., McKiernan J.M. (2008). Epidemiology, Clinical Staging, and Presentation of Renal Cell Carcinoma. Urol. Clin. N. Am..

[5] Creighton C.J., Hernandez-Herrera A., Jacobsen A., Levine D.A., Mankoo P., Schultz N., Du Y., Zhang Y., Larsson E., Sheridan R. (2012). Integrated Analyses of MicroRNAs Demonstrate Their Widespread Influence on Gene Expression in High-Grade Serous Ovarian Carcinoma. PLoS ONE.

[6] Scelo G., Larose T.L. (2018). Epidemiology and Risk Factors for Kidney Cancer. J. Clin. Oncol..

[7] Howlader N., Noone A.M., Krapcho M., Miller D., Brest A., Yu M., Ruhl J., Tatalovich Z., Mariotto A., Lewis D.R. (2019). SEER Cancer Statistics Review, 1975–2016.

[8] van de Pol J.A.A., George L., van den Brandt P.A., Baldewijns M.M.L.L., Schouten L.J. (2021). Etiologic Heterogeneity of Clear-Cell and Papillary Renal Cell Carcinoma in the Netherlands Cohort Study. Int. J. Cancer.

[9] Kase A.M., George D.J., Ramalingam S. (2023). Clear Cell Renal Cell Carcinoma: From Biology to Treatment. Cancers.

[10] Morgan T.M., Seeley E.H., Fadare O., Caprioli R.M., Clark P.E. (2013). Imaging the Clear Cell Renal Cell Carcinoma Proteome. J. Urol..

[11] Chinello C., L’Imperio V., Stella M., Smith A.J., Bovo G., Grasso A., Grasso M., Raimondo F., Pitto M., Pagni F. (2016). The Proteomic Landscape of Renal Tumors. Expert Rev. Proteom..

[12] Song Y., Zhong L., Zhou J., Lu M., Xing T., Ma L., Shen J. (2017). Data-Independent Acquisition-Based Quantitative Proteomic Analysis Reveals Potential Biomarkers of Kidney Cancer. Proteom. Clin. Appl..

[13] Mertins P., Mani D.R., Ruggles K.V., Gillette M.A., Clauser K.R., Wang P., Wang X., Qiao J.W., Cao S., Petralia F. (2016). Proteogenomics Connects Somatic Mutations to Signalling in Breast Cancer. Nature.

[14] Zhang B., Wang J., Wang X., Zhu J., Liu Q., Shi Z., Chambers M.C., Zimmerman L.J., Shaddox K.F., Kim S. (2014). Proteogenomic Characterization of Human Colon and Rectal Cancer. Nature.

[15] Clark D.J., Dhanasekaran S.M., Petralia F., Pan J., Song X., Hu Y., da Veiga Leprevost F., Reva B., Lih T.S.M., Chang H.Y. (2019). Integrated Proteogenomic Characterization of Clear Cell Renal Cell Carcinoma. Cell.

[16] Jara J.H., Frank D.D., Özdinler P.H. (2013). Could Dysregulation of UPS Be a Common Underlying Mechanism for Cancer and Neurodegeneration? Lessons from UCHL1. Cell Biochem. Biophys..

[17] Matuszczak E., Tylicka M., Dębek W., Tokarzewicz A., Gorodkiewicz E., Hermanowicz A. (2018). Concentration of UHCL1 in the Serum of Children with Acute Appendicitis, Before and After Surgery, and Its Correlation with CRP and Prealbumin. J. Investig. Surg..

[18] Kastner B., Will C.L., Stark H., Lührmann R. (2019). Structural Insights into Nuclear Pre-MRNA Splicing in Higher Eukaryotes. Cold Spring Harb. Perspect. Biol..

[19] Jiménez-Vacas J.M., Herrero-Aguayo V., Montero-Hidalgo A.J., Gómez-Gómez E., Fuentes-Fayos A.C., León-González A.J., Sáez-Martínez P., Alors-Pérez E., Pedraza-Arévalo S., González-Serrano T. (2020). Dysregulation of the Splicing Machinery Is Directly Associated to Aggressiveness of Prostate Cancer. eBioMedicine.

[20] Jaffer Z.M., Chernoff J. (2002). P21-Activated Kinases: Three More Join the Pak. Int. J. Biochem. Cell Biol..

[21] Dummler B., Ohshiro K., Kumar R., Field J. (2009). Pak Protein Kinases and Their Role in Cancer. Cancer Metastasis Rev..

[22] Hirsch F.R., Varella-Garcia M., Bunn P.A., Di Maria M.V., Veve R., Bremnes R.M., Barón A.E., Zeng C., Franklin W.A. (2003). Epidermal Growth Factor Receptor in Non-Small-Cell Lung Carcinomas: Correlation between Gene Copy Number and Protein Expression and Impact on Prognosis. J. Clin. Oncol..

[23] Wang X., Zhang N., Li M., Hong T., Meng W., Ouyang T. (2023). Ubiquitin C-terminal Hydrolase-L1: A New Cancer Marker and Therapeutic Target with Dual Effects (Review). Oncol. Lett..

[24] Li L., Tao Q., Jin H., Van Hasselt A., Poon F.F., Wang X., Zeng M.S., Jia W.H., Zeng Y.X., Chan A.T.C. (2010). The Tumor Suppressor UCHL1 Forms a Complex with P53/MDM2/ARF to Promote P53 Signaling and Is Frequently Silenced in Nasopharyngeal Carcinoma. Clin. Cancer Res..

[25] Wang G., Zhang W., Zhou B., Jin C., Wang Z., Yang Y., Wang Z., Chen Y., Feng X. (2015). The Diagnosis Value of Promoter Methylation of UCHL1 in the Serum for Progression of Gastric Cancer. BioMed Res. Int..

[26] Jin C., Yu W., Lou X., Zhou F., Han X., Zhao N., Lin B. (2013). UCHL1 Is a Putative Tumor Suppressor in Ovarian Cancer Cells and Contributes to Cisplatin Resistance. J. Cancer.

[27] The Human Protein Atlas. https://www.proteinatlas.org/.

[28] Takano T., Miyauchi A., Matsuzuka F., Yoshida H., Nakata Y., Kuma K., Amino N. (2004). PGP9.5 MRNA Could Contribute to the Molecular-Based Diagnosis of Medullary Thyroid Carcinoma. Eur. J. Cancer.

[29] Kagara I., Enokida H., Kawakami K., Matsuda R., Toki K., Nishimura H., Chiyomaru T., Tatarano S., Itesako T., Kawamoto K. (2008). CpG Hypermethylation of the UCHL1 Gene Promoter Is Associated with Pathogenesis and Poor Prognosis in Renal Cell Carcinoma. J. Urol..

[30] Seliger B., Handke D., Schabel E., Bukur J., Lichtenfels R., Dammann R. (2009). Epigenetic Control of the Ubiquitin Carboxyl Terminal Hydrolase 1 in Renal Cell Carcinoma. J. Transl. Med..

[31] Kim H.J., Kim Y.M., Lim S., Nam Y.K., Jeong J., Kim H.J., Lee K.J. (2009). Ubiquitin C-Terminal Hydrolase-L1 Is a Key Regulator of Tumor Cell Invasion and Metastasis. Oncogene.

[32] Hussain S., Foreman O., Perkins S.L., Witzig T.E., Miles R.R., Van Deursen J., Galardy P.J. (2010). The De-Ubiquitinase UCH-L1 Is an Oncogene That Drives the Development of Lymphoma in Vivo by Deregulating PHLPP1 and Akt Signaling. Leukemia.

[33] Hennessy B.T., Smith D.L., Ram P.T., Lu Y., Mills G.B. (2005). Exploiting the PI3K/AKT Pathway for Cancer Drug Discovery. Nat. Rev. Drug Discov..

[34] Yoeli-Lerner M., Toker A. (2006). Akt/PKB Signaling in Cancer: A Function in Cell Motility and Invasion. Cell Cycle.

[35] Harada T., Harada C., Wang Y.L., Osaka H., Amanai K., Tanaka K., Takizawa S., Setsuie R., Sakurai M., Sato Y. (2004). Role of Ubiquitin Carboxy Terminal Hydrolase-L1 in Neural Cell Apoptosis Induced by Ischemic Retinal Injury in Vivo. Am. J. Pathol..

[36] Mani A., Gelmann E.P. (2005). The Ubiquitin-Proteasome Pathway and Its Role in Cancer. J. Clin. Oncol..

[37] Ummanni R., Jost E., Braig M., Lohmann F., Mundt F., Barett C., Schlomm T., Sauter G., Senff T., Bokemeyer C. (2011). Ubiquitin Carboxyl-Terminal Hydrolase 1 (UCHL1) Is a Potential Tumour Suppressor in Prostate Cancer and Is Frequently Silenced by Promoter Methylation. Mol. Cancer.

[38] Cheng J., Liu H., Shen Y., Ding J., He H., Mao S., Chen L., Zhang C., Zhou J. (2024). Deubiquitinase UCHL1 Stabilizes KDM4B to Augment VEGF Signaling and Confer Bevacizumab Resistance in Clear Cell Renal Cell Carcinoma. Transl. Oncol..

[39] Gottschalk A., Kastner B., Lührmann R., Fabrizio P. (2001). The Yeast U5 SnRNP Coisolated with the U1 SnRNP Has an Unexpected Protein Composition and Includes the Splicing Factor Aar2p. RNA.

[40] Ye Z., Bing A., Zhao S., Yi S., Zhan X. (2022). Comprehensive Analysis of Spliceosome Genes and Their Mutants across 27 Cancer Types in 9070 Patients: Clinically Relevant Outcomes in the Context of 3P Medicine. EPMA J..

[41] Zhan Y.T., Li L., Zeng T.T., Zhou N.N., Guan X.Y., Li Y. (2021). SNRPB-Mediated RNA Splicing Drives Tumor Cell Proliferation and Stemness in Hepatocellular Carcinoma. Aging.

[42] Li X., Turanli B., Juszczak K., Kim W., Arif M., Sato Y., Ogawa S., Turkez H., Nielsen J., Boren J. (2020). Classification of Clear Cell Renal Cell Carcinoma Based on PKM Alternative Splicing. Heliyon.

[43] Zhang T., Bai J., Zhang X., Zheng X., Lu N., Liang Z., Lin L., Chen Y. (2021). SNRNP200 Mutations Cause Autosomal Dominant Retinitis Pigmentosa. Front. Med..

[44] Knorr K., Rahman J., Erickson C., Wang E., Monetti M., Li Z., Ortiz-Pacheco J., Jones A., Lu S.X., Stanley R.F. (2023). Systematic Evaluation of AML-Associated Antigens Identifies Anti-U5 SNRNP200 Therapeutic Antibodies for the Treatment of Acute Myeloid Leukemia. Nat. Cancer.

[45] Bradley R.K., Anczuków O. (2023). RNA Splicing Dysregulation and the Hallmarks of Cancer. Nat. Rev. Cancer.

[46] Ivanova O.M., Anufrieva K.S., Kazakova A.N., Malyants I.K., Shnaider P.V., Lukina M.M., Shender V.O. (2023). Non-Canonical Functions of Spliceosome Components in Cancer Progression. Cell Death Dis..

[47] Kichina J.V., Goc A., Al-Husein B., Somanath P.R., Kandel E.S. (2010). PAK1 as a Therapeutic Target. Expert Opin. Ther. Targets.

[48] Ye D.Z., Field J. (2012). PAK Signaling in Cancer. Cell. Logist..

[49] Yeo D., He H., Baldwin G.S., Nikfarjam M. (2015). The Role of P21-Activated Kinases in Pancreatic Cancer. Pancreas.

[50] Liu H., Liu K., Dong Z. (2021). The Role of P21-Activated Kinases in Cancer and Beyond: Where Are We Heading?. Front. Cell Dev. Biol..

[51] Kumar R., Sanawar R., Li X., Li F. (2017). Structure, Biochemistry, and Biology of PAK Kinases. Gene.

[52] O’Sullivan G.C., Tangney M., Casey G., Ambrose M., Houston A., Barry O.P. (2007). Modulation of P21-Activated Kinase 1 Alters the Behavior of Renal Cell Carcinoma. Int. J. Cancer.

[53] Liu W., Yang Y., Liu Y., Liu H., Zhang W., Xu L., Zhu Y., Xu J. (2015). P21-Activated Kinase 4 Predicts Early Recurrence and Poor Survival in Patients with Nonmetastatic Clear Cell Renal Cell Carcinoma. Urol. Oncol. Semin. Orig. Investig..

[54] Kang H.W., Piao X.M., Lee H.Y., Kim K., Seo S.P., Ha Y.S., Kim Y.U., Kim W.T., Kim Y.J., Lee S.C. (2021). Expression of Phosphorylated P21-Activated Kinase 4 Is Associated with Aggressive Histologic Characteristics and Poor Prognosis in Patients with Surgically Treated Renal Cell Carcinoma. Investig. Clin. Urol..

[55] Linehan W.M., Ricketts C.J. (2019). The Cancer Genome Atlas of Renal Cell Carcinoma: Findings and Clinical Implications. Nat. Rev. Urol..

[56] Park J.J., Park M.H., Oh E.H., Soung N.K., Lee S.J., Jung J.K., Lee O.J., Yun S.J., Kim W.J., Shin E.Y. (2018). The P21-Activated Kinase 4-Slug Transcription Factor Axis Promotes Epithelial−mesenchymal Transition and Worsens Prognosis in Prostate Cancer. Oncogene.

[57] Zhang J., Wang J., Guo Q., Wang Y., Zhou Y., Peng H., Cheng M., Zhao D., Li F. (2012). LCH-7749944, a Novel and Potent P21-Activated Kinase 4 Inhibitor, Suppresses Proliferation and Invasion in Human Gastric Cancer Cells. Cancer Lett..

[58] Ryu B.J., Lee H., Kim S.H., Heo J.N., Choi S.W., Yeon J.T., Lee J., Lee J., Cho J.Y., Kim S.H. (2014). PF-3758309, P21-Activated Kinase 4 Inhibitor, Suppresses Migration and Invasion of A549 Human Lung Cancer Cells via Regulation of CREB, NF-ΚB, and β-Catenin Signalings. Mol. Cell. Biochem..

[59] Li Z., Li X., Xu L., Tao Y., Yang C., Chen X., Fang F., Wu Y., Ding X., Zhao H. (2017). Inhibition of Neuroblastoma Proliferation by PF-3758309, a Small-Molecule Inhibitor That Targets P21-Activated Kinase 4. Oncol. Rep..

