# Supplementary material for: Prognostic Significance of Elevated UCHL1, SNRNP200, and PAK4 Expression in High-Grade Clear Cell Renal Cell Carcinoma: Insights from LC-MS/MS Analysis and Immunohistochemical Validation
Source: Cancers (Basel). 2024 Aug 14;16(16):2844. doi: 10.3390/cancers16162844 (PMC11352290; doi:10.3390/cancers16162844)
Supplement: Supplementary file 1 [file cancers-16-02844-s001.zip › cancers-3124929-supplementary.pdf]

Table S1. Characteristics of patients included in the study.

NO.	AGE AT PRIMARY TUMOR SURGERY	HISTOLOGY GRADE	METASTASIS LOCATION	PFS (DAYS)	OS (DAYS)	PATIENT STATUS
**1**	60	G3	No metastasis	1920	2226	Alive
**2**	84	G1	No metastasis	1637	1637	Deceased
**3**	68	G2	No metastasis	1898	2204	Alive
**4**	31	G3	No metastasis	1888	2194	Alive
**5**	78	G2	Liver	15	140	Deceased
**6**	68	G2	No metastasis	1870	2176	Alive
**7**	67	G3	No metastasis	1856	2162	Alive
**8**	69	G2	No metastasis	1644	1644	Deceased
**9**	54	G3	No metastasis	112	113	Deceased
**10**	49	G2	No metastasis	1801	2107	Alive
**11**	66	G3	No metastasis	1793	2099	Alive
**12**	52	G2	Lungs	38	455	Deceased
**13**	79	G2	No metastasis	1778	2084	Alive
**14**	65	G2	No metastasis	1769	2075	Alive
**15**	60	G1	No metastasis	1724	2030	Alive
**16**	71	G2	Lungs	14	2030	Alive
**17**	58	G2	No metastasis	1716	2022	Alive
**18**	72	G2	No metastasis	1824	2008	Alive
**19**	51	G2	No metastasis	1824	2008	Alive
**20**	48	G2	No metastasis	1819	2002	Alive
**21**	69	G2	No metastasis	1647	1953	Alive
**22**	51	G2	Liver	9	1952	Alive
**23**	58	G2	Lung	362	1950	Alive
**24**	64	G3	Liver	1220	1918	Alive
**25**	64	G2	Kidney	862	1909	Alive
**26**	68	G2	Lung	545	1896	Alive
**27**	74	G2	No metastasis	1589	1895	Alive
**28**	59	G1	No metastasis	1567	1873	Alive
**29**	67	G2	Lung	144	1857	Alive
**30**	69	G2	No metastasis	1552	1858	Alive
**31**	68	G2	Kidney	14	1854	Alive
**32**	56	G1	No metastasis	1542	1848	Alive
**33**	65	G2	No metastasis	1428	1734	Alive
**34**	46	G2	No metastasis	1428	1734	Alive
**35**	65	G1	No metastasis	1217	1217	Deceased
**36**	50	G3	No metastasis	1420	1726	Alive
**37**	62	G3	Lungs	0	1187	Alive
**38**	50	G3	Lungs	659	1169	Alive
**39**	62	G3	Lungs	192	437	Deceased
**40**	42	G2	Lungs, liver, kidney	127	144	Deceased
**41**	80	G2	Lungs, liver, kidney	448	541	Deceased
**42**	58	G1	Lungs, liver, kidney	335	424	Deceased
**43**	56	G3	Lungs	49	1544	Deceased
**44**	54	G1	Lungs, kidney	327	2878	Alive
**45**	52	G3	Liver, bones	0	101	Deceased
**46**	57	G2	Lungs	0	876	Deceased
**47**	55	G3	Lungs	1199	2309	Alive
**48**	56	G2	Kidney	2093	2462	Alive
**49**	50	G2	Lungs	681	2394	Alive
**50**	57	G3	Lungs	51	1212	Deceased
**51**	51	G3	Bones	662	731	Deceased
**52**	52	G2	Bones	174	1341	Alive
